# A Novel Single-Sample Retinal Vessel Segmentation Method Based on Grey Relational Analysis

**DOI:** 10.3390/s24134326

**Published:** 2024-07-03

**Authors:** Yating Wang, Hongjun Li

**Affiliations:** School of Information Science and Technology, Nantong University, Nantong 226019, China; 2330310035@stmail.ntu.edu.cn

**Keywords:** retinal vessel segmentation, grey relational analysis, adaptive discriminative filtering, unsupervised algorithm

## Abstract

Accurate segmentation of retinal vessels is of great significance for computer-aided diagnosis and treatment of many diseases. Due to the limited number of retinal vessel samples and the scarcity of labeled samples, and since grey theory excels in handling problems of “few data, poor information”, this paper proposes a novel grey relational-based method for retinal vessel segmentation. Firstly, a noise-adaptive discrimination filtering algorithm based on grey relational analysis (NADF-GRA) is designed to enhance the image. Secondly, a threshold segmentation model based on grey relational analysis (TS-GRA) is designed to segment the enhanced vessel image. Finally, a post-processing stage involving hole filling and removal of isolated pixels is applied to obtain the final segmentation output. The performance of the proposed method is evaluated using multiple different measurement metrics on publicly available digital retinal DRIVE, STARE and HRF datasets. Experimental analysis showed that the average accuracy and specificity on the DRIVE dataset were 96.03% and 98.51%. The mean accuracy and specificity on the STARE dataset were 95.46% and 97.85%. Precision, F1-score, and Jaccard index on the HRF dataset all demonstrated high-performance levels. The method proposed in this paper is superior to the current mainstream methods.

## 1. Introduction

In recent years, the prevalence of diseases such as diabetic retinopathy and hypertension has been on the rise due to irregular lifestyles and unhealthy eating habits. According to the International Diabetes Federation’s latest estimates for 2021, approximately 537 million people worldwide are living with diabetes [1]. In most cases, retinal diseases are asymptomatic until they reach an advanced stage. Therefore, timely detection and treatment are essential to prevent irreversible blindness. Therefore, accurate segmentation of retinal blood vessels is a key task in diagnosing these diseases.

**Figure 1:**
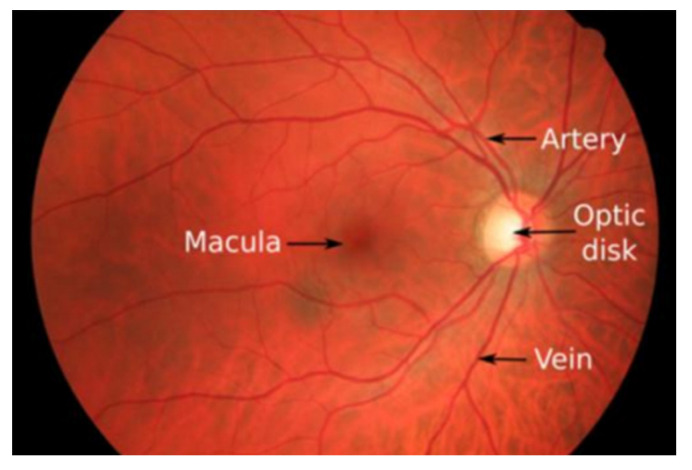
Structure of the retina.

The eyeball wall is mainly composed of three layers: the inner, middle, and outer layers. The innermost layer, known as the retina, is located in the innermost part of the eyeball. It contains many light-sensitive cells responsible for photoreception, converting light signals into visual signals that are transmitted to the brain’s visual center to form images. The retina is the most sensitive area for neural information transmission. The structure of the retina includes the optic disc, macula, fovea, and retinal blood vessels, as shown in Figure 1. The retinal vascular tree consists of the central artery, veins, and their branches. Abnormalities may include microaneurysms, hemorrhages, exudates, and cotton wool spots. The fundus is the only place where arteries, veins, and capillaries can be directly observed, reflecting the dynamic and health status of the entire body’s blood circulation. Additionally, the retina is the only part of the human body where its anatomical structure can be visualized directly and non-invasively. Retinal vessel segmentation technology provides important information such as the shape, thickness, and curvature of retinal blood vessels.

A challenging problem in retinal vessel segmentation is to establish a general segmentation algorithm that is robust enough to the various types of noise that may occur. Generally, the algorithms can broadly be categorized into unsupervised methods and supervised methods. With the development of deep learning, there are many new research results categorized as supervised methods. Tang et al. [2] proposed a multi-proportional channel and U-Net ensemble model for blood vessel extraction. Guo et al. [3] introduced a high-resolution hierarchical network to identify and classify features. Huang et al. [4] proposed a cascade self-attention U-net for accurate segmentation of retinal blood vessels. Xie et al. [5] proposed a method that combines optical coherence tomography angiography (OCTA) with deep learning to achieve high-precision automatic segmentation and parameter extraction of retinal microvasculature and the foveal avascular zone (FAZ). Using these parameters, they explored their potential value in predicting Alzheimer’s Disease (AD) and mild cognitive impairment (MCI). Meng et al. [6] proposed a Dual Adaptive Graph Convolutional Network (DAGCN) for weakly and semi-supervised segmentation of the optic disc and cup, leveraging geometric associations and dual consistency regularization for enhanced performance in glaucoma assessment. This model demonstrates excellent performance on OD&OC segmentation and vCDR estimation, which is of significant importance for glaucoma screening and evaluation. Hao et al. [7] proposed a Voting-based Adaptive Feature Fusion multi-task network (VAFF-Net) for the simultaneous segmentation, detection, and classification of retinal structures in OCTA images, enhancing the precision of ophthalmic diagnostics. Xia et al. [8] proposed an edge-reinforced network (ER-Net) for the segmentation of 3D vessel-like structures in medical images, incorporating a reverse edge attention module (REAM), a feature selection module (FSM), and an edge-reinforced loss function to enhance the delineation of crisp edges and improve segmentation accuracy. Zhao et al. [9] proposed a novel 2-D/3-D symmetry filter for the enhancement of vascular structures in multiple modality images, leveraging a weighted geometric mean approach to address challenges posed by imaging artifacts, contrast variations, and noise, thereby improving the detection and segmentation of blood vessels. Ma et al. [10] introduced a novel split-based coarse-to-fine vessel segmentation network for Optical Coherence Tomography Angiography (OCTA) images, termed OCTA-Net, which employs a ResNeSt backbone and a two-stage training approach to enhance the segmentation of both thick and thin retinal vessels effectively. Despite their effectiveness, these methods often require extensive training datasets, which are computationally demanding to build. In addition, the number of retinal blood vessel samples is limited and labeled samples are even more scarce, and the extraction of blood vessel samples requires certain medical knowledge, usually requiring professional medical personnel to label, which is a time-consuming and laborious process.

Single-sample retinal vessel segmentation still holds significant research significance. This unsupervised learning approach extracts vessel and background features from fundus images without relying on label information, identifying their relationship to achieve vessel segmentation. There have been many classical methods for vessel segmentation, such as traditional matched filtering, vessel tracking, multiscale segmentation, morphological-based methods, and thresholding methods. Zhang et al. [11] proposed an MF-FDOG blood vessel extraction method combining matched filtering and Gaussian first derivative. This method does not guarantee good vascular connectivity. Krause et al. [12] used the local Radon transform method, which had lower computational cost but poor performance in the segmentation of small blood vessels. Zhao et al. [13] proposed a segmentation method based on level set and region growth. Because the active contours tend to move towards the diseased area, segmentation of some abnormal retinal images is inaccurate.

This study focuses on single retinal vessel images, considering the limited sample size and associated uncertainty. To address the small sample problem and enhance the overall algorithm’s specificity and accuracy, gray theory is introduced. These improvements are not merely descriptive but are substantiated with theoretical foundations and demonstrate their effectiveness through experiments. The refinements introduced aim to significantly enhance the robustness and reliability of retinal vessel analysis in medical imaging applications. This study provides a new theoretical framework and algorithm tool for small sample image analysis. The main contributions of this paper are as follows:

(1) In order to effectively reduce noise interference and enhance the pre-processing stage to achieve accurate retinal blood vessel segmentation, a noise-adaptive discrimination filtering algorithm based on grey relational analysis is proposed. It adaptively adjusts the filtering intensity through the gray correlation degree to distinguish between noise and real features, ensuring that the key vascular structure is preserved without being obscured by noise. This approach retains more critical details while achieving improved filtering effects.
(2) A threshold segmentation model based on grey relational analysis is proposed to accurately localize the direction of vessels. This model can not only detect retinal blood vessels effectively but also reduce the interference of abnormal retinal noise signals to a certain extent. The study discusses in depth the theoretical underpinning of the model, including how grey correlation analysis quantifies the differences between blood vessels and the background, and provides an evaluation of the model’s performance in actual image segmentation.

## 2. Proposed Method

Grey system theory was originally proposed by Deng Julong at Huazhong University of Science and Technology in 1980 [14]. This theory was developed to address the issue of “few data, poor information”. In scientific research, people usually use “black” to mean that the information is completely unknown, and “white” to mean that the information is completely known. “Gray” means that part of the information is unknown, and part is known, so these information systems are called “gray systems”. Grey relational analysis is a mathematical theory that can quantitatively obtain unknown information by referring to known information. Its essence is to judge the degree of correlation between the sequences according to the similarity of the sequences. Compared with traditional methods, grey relational model has the following advantages: it does not require a large number of sample data; the specific statistical laws of the system do not need to be known; there is no need to consider the independence of each factor. In recent years, grey relational analysis has been gradually applied to image processing, and the effect is remarkable. Ma et al. [15] successfully integrated Deng’s traditional correlation coefficient into an image edge detection algorithm, effectively merging grey relational analysis with edge detection and yielding optimal results experimentally. Zhen et al. [16] combined grey relational analysis with genetic algorithms to effectively segment target regions, demonstrating certain noise resistance. Li et al. [17] proposed an image edge detection algorithm based on grey simplified B-type relational analysis and realized optimal threshold selection through iteration. The algorithm exhibits strong adaptability to images with drastic grayscale changes, resulting in clear and accurate edge detection. These algorithms make reasonable use of various forms of grey relational analysis for image edge detection, effectively detecting texture edges.

In grey relational analysis, the most classic and widely used is the traditional Deng correlation degree. First of all, in order to standardize the data dimension of each sequence and enhance the comparability of data, it is generally necessary to carry out data normalization processing. Then, the system feature sequence (reference sequence) and related factor sequence (comparison sequence) are established. Then the grey correlation degree and coefficient are obtained.

**Figure 2:**
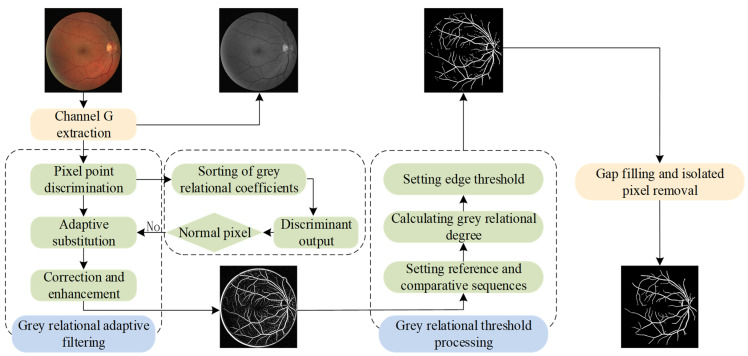
Block diagram of the suggested approach.

Considering that the labeling samples of retinal blood vessel images are few, there are uncertain noise and other factors. In this paper, the advantages of a grey system in uncertainty and a small sample problem are used to propose a novel single-sample retinal vessel segmentation method based on grey relational analysis. Figure 2 illustrates the flowchart of the proposed method. First of all, the noise is discrete, and the traditional grey correlation filtering algorithm cannot be used to unify all pixels; otherwise, the real pixel gray scale will be lost. In order to avoid false filtering, a novel noise-adaptive discrimination filtering algorithm based on grey relational analysis is proposed, and a good filtering effect is achieved. Secondly, a threshold segmentation model based on grey relational coefficients is proposed to improve the traditional grey correlation degree model, which avoids the pathological situation that the denominator may be zero in the Dunn correlation degree, and eliminates the normalization process in the traditional Dunn correlation degree calculation, which enhances the stability and executability of the model and achieves good operation results.

### 2.1. Noise-Adaptive Discrimination Filtering Algorithm Based on Grey Relational Analysis (NADF-GRA)

Since image noise typically consists of high-frequency components, the gray value of a noisy pixel tends to be at or near the extreme values within the filtering window. If the image correlation coefficient between the center pixel of the window and the median value of the domain is small, the center pixel is considered to be a pixel that deviates from the value of the neighborhood and can be identified as noise. Otherwise, it is considered a normal pixel. In the final filtering, only the normal pixels in the marker matrix are selected for grey correlation weighted mean filtering. If the flag matrix displays all noise points, the filtering window can be extended for filtering. Since the effective pixel points when the window is expanded are all points away from the center pixel, it can be changed to nonlinear filtering at this time, which can be replaced by a simple median filter, so that a good filtering effect can be achieved. The filtering algorithm commences by distinguishing noise from normal pixels. This step is critical as it lays the foundation for the subsequent application of our weighted averaging technique. By applying weights, normal pixels that are critical to vascular structure are given higher significance, thus preserving the integrity of retinal blood vessels. And the adaptive nature of our weighted scheme allows for the effective suppression of noise identified during the initial filtering stage, without compromising the vascular features. The dynamic weighting of pixels in our algorithm ensures robust performance across images with varying noise characteristics, maintaining consistent segmentation results.

The algorithm implementation process can be divided into three stages: Firstly, the discrimination stage utilizes grey relational coefficients for noise determination, which provides relevant records distinguishing normal pixels from noise pixels. Secondly, the adaptive adjustment of pixels, where weighted averaging filtering is applied based on the acquired information of normal pixels, thereby eliminating the interference of noise pixels. If there are no normal pixels within the filtering window, consideration is given to expanding the filtering window for simple median filtering. Lastly, the calibration and enhancement module.

In the 3 × 3 filtering window as the central pixel, the median pixel value in the field is selected as the reference sequence, and the 9-pixel value in the field is selected as the comparison sequence:

(1) $$X_{0}=\{x_{0}(1),...,x_{0}(9)\}=\{v,...,v\}$$

(2) $$X_{1}=\{f(i-1,j-1),...,f(i+1,j+1)\}$$

where $X_{0}$ is the reference sequence, $X_{1}$ is the comparison sequence, $x_{0}(1),...,x_{0}(9)$ is the 9-pixel value of the reference sequence, v is the median pixel in the field and f(i−1,j−1),...,f(i+1,j+1) is the 9-pixel value of the comparison sequence.

To calculate the image grey relational coefficients between the median value of the filtering window and the values of each pixel in the neighborhood:

(3) $$\gamma_{k}=\gamma (x_{0}(k),x_{1}(k))=\frac{1}{1+\left|x_{0}(k)-x_{1}(k)\right|}$$

where $\gamma_{k}$ is the image association coefficient, $x_{0}(k)$ is the median value in the domain, and $x_{1}(k)$ is each value in the domain.

The image association coefficients calculated above are sorted in order from small to large to obtain the gray image association order.

Test whether the gray image association coefficient of the center pixel of the filtering window is ranked in the first three in the association order: If the association coefficient of the center pixel is ranked in the first three, it indicates that the gray value deviates from the median value of the domain, set T(i,j)=0, and mark it as a noise pixel; otherwise, set T(i,j)=1, and mark it as a normal pixel.

(4) $$T(i,j)=\left\{\begin{array}{c} 0\;\;\mathrm{if}\;\gamma_{5}=\varepsilon_{1}\;\mathrm{or}\;\gamma_{5}=\varepsilon_{2}\;\mathrm{or}\;\gamma_{5}=\varepsilon_{3}\;\;\;\;\;\;\;\;\;\;\\ 1\;\;\;\;\;\;\;\;\;\;\;\;\;\;\;\mathrm{otherwise}\;\;\;\;\;\;\;\;\;\;\;\;\;\;\;\;\;\;\;\;\;\;\;\;\;\;\;\;\;\;\;\;\;\;\;\; \end{array}\right.$$

where T(i,j) is the flag matrix, $\gamma_{5}$ is the gray image association coefficient of the center pixel, and $\varepsilon_{1}{,\varepsilon }_{2},\varepsilon_{3}$ is the coefficient value of the first three sorted in the association order.

From top to bottom and from left to right, each pixel in the image is iterated in turn so that a matrix T with element 0 or 1 marking the image noise information can be obtained.

The above is the noise detection stage of the image, and the next step is the noise point replacement stage of the image: starting from the upper left corner of the image, check whether the corresponding element in the flag matrix T(i,j) corresponding to the center pixel f(i,j) of the filtering window is equal to 1; if it is equal to 1, the current pixel is a normal pixel point, the pixel value remains unchanged, and the loop enters the next pixel for judgment; if it is equal to 0, it means that the corresponding point in the image is a noise point and should be filtered: at this time, the number of normal pixels in the 3 × 3 window field with f(i,j) as the center pixel should be calculated, denoted as C; if C > 0, the C pixel is taken as the comparison sequence, where the value is the reference sequence, the image association coefficient is calculated, and the weighted mean of the grey association is filtered. At this time, there is:

(5) $$f(i,j)=(\sum_{h=1}^{C}\gamma_{h}\cdot f_{h})/\sum_{h=1}^{C}\gamma_{h}$$

where f(i,j) is the central pixel, C is the number of normal pixels in the field, $\gamma_{h}$ is the image association coefficient, and $f_{h}$ is the pixel in the field. If C = 0, it means that all the pixels in the 3 × 3 window are polluted by noise, which is a large noise block. At this time, the filtering window should be expanded into a 5 × 5 filtering window because the outermost non-noisy pixels in the window are already far away from the center pixel. Therefore, you can simply use the median filter to complete the assignment of the center pixel. Each pixel is processed in the traversal order from top to bottom and from left to right throughout the program loop.

Finally, it enters the adaptive correction and enhancement stage: contrast adaptive histogram equalization is used to effectively improve image contrast, and the similarity between image regions and blood vessels is evaluated on different scales to enhance the detection accuracy of blood vessel structure.

### 2.2. Threshold Segmentation Model Based on Grey Relational Analysis (TS-GRA)

The basic idea of grey relational analysis is to measure the similarity between reference sequence and comparison sequence by grey relational degree. The edge of the image generally has grayscale mutations to a certain extent, and the grayscale of these mutated pixels generally maintains continuity in a specific direction or texture pattern, and the gray correlation degree can just reflect the degree of such mutations. When detecting the edge of the image, set the mean value of the pixels in the field as the reference sequence. If the comparison sequence is farther away from the reference sequence, it indicates that the image has edge passing in the field, and the grey correlation degree value will be smaller. On the contrary, if no edge passes through, the value of the gray correlation degree will be greater at this time. By setting a threshold, edges can be found in the image.

Set the current center pixel in the 3×3 domain window of the image as f(i,j)(i=2,3,...,M−1;j=2,3,...,N−1), first calculate the mean value of all pixels in the domain window, and then set the reference sequence and comparison sequence. 

The difference sequence between the reference sequence and the comparison sequence can be obtained as follows:

(6) $$\Delta (u)=\left|x_{0}(u)-x_{1}(u)\right|,u=1,2,...,9$$

where Δ(u) is the difference sequence, $x_{0}(u)$ is the reference sequence, and $x_{1}(u)$ is the comparison sequence.

Calculate the grey image correlation coefficient:

(7) $$\gamma_{01}(u)=\frac{1}{1+\Delta (u)}$$

where $\gamma_{01}(u)$ is the correlation coefficient of the gray image of each pixel, and Δ(u) is the difference sequence.

Calculate the gray image correlation degree of the center pixel f(i,j) of the image domain window:

(8) $$\gamma_{01}(i,j)=\frac{1}{9}\sum_{u=1}^{9}\gamma_{01}(u)$$

where $\gamma_{01}(i,j)$ is the gray image correlation degree of the center pixel in the domain, and $\gamma_{01}(u)$ is the relational coefficient of each point.

The first four steps start at the top left corner of the image, proceeding in order from left to right and top to bottom, saving the corresponding grey relational coefficients for each pixel as the center pixel of the domain window in a table until the last pixel in the bottom right corner of the image is traversed.

Find the minimum and maximum values of the grey relational coefficients corresponding to all pixels. Establish a threshold value between the minimum and maximum values as the threshold for distinguishing whether the current point is an edge point or a non-edge point, that is:

(9) $$Tag(i,j)=\left\{\begin{array}{c} 1,\gamma_{01}(i,j)<\theta \\ 0,\;\;\;\;\mathrm{otherwise} \end{array}\right.$$

where θ is the threshold to distinguish whether it is an edge point, and Tag(i,j) is the matrix that stores whether it is an edge point.

The final segmentation results are obtained through the post-processing stage using morphological manipulation. A disc-shaped structural element was created for closure operations to fill the void in the blood vessel and connect the broken blood vessel, thereby improving vascular connectivity. Removing small, connected areas, thereby eliminating error-detected independent pixels, helps remove noise or pseudo-targets.

### 2.3. Contrast Adaptive Histogram Equalization (CLAHE)

Intensity inhomogeneity is usually generated during retinal image acquisition, for which it is necessary to perform image enhancement or inhomogeneity correction to eliminate the effect under different lighting conditions. Histogram equalization, a global enhancement method that takes into account the color frequency and intensity of pixels in an image and then redistributes these properties, is effective for images where color and intensity are concentrated in a narrow band. However, it cannot handle images with colors and intensities that span the entire range of display devices. Another widely adopted global enhancement method is gamma correction, which applies a nonlinear function to the pixel values of the input image to adjust the brightness of the image, making dark areas brighter and bright areas darker to improve the contrast and detail of the image. However, the best choice for parameter gamma depends on the image under consideration. Contrast-limited adaptive histogram equalization (CLAHE) divides the entire image space into several small, equally sized regions and processes each region individually, where the contrast of each small region increases so that the histogram of the output image corresponds to the histogram indicated by the distribution parameters. The adjacent small regions are then combined using bilinear interpolation, which suppresses artificially set limits. By limiting the contrast of a single uniform area, it is possible to avoid uneven areas in retinal image analysis, while also avoiding excessive noise amplification.

### 2.4. Frangi Filtering

**Figure 3:**
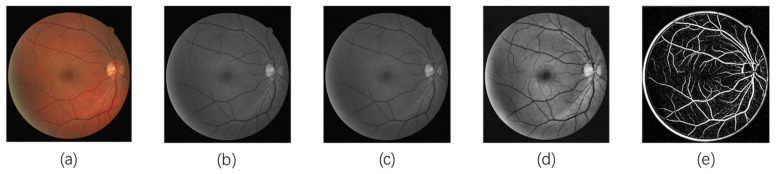
Middle image of fundus image preprocessing: (**a**) the original image, (**b**) the green channel image, (**c**) the image after NADF-GRA, (**d**) the image after CLAHE, (**e**) the image after Frangi enhancement.

A Frangi filter is a filter used to enhance the structure of blood vessels, which is calculated based on the eigenvalues and eigenvectors of the Hessian matrix. Its main principle is to calculate the Hessian matrix on different scales and evaluate the similarity between the image region and the blood vessels by calculating the eigenvalues and eigenvectors. In addition, the Frangi filter also takes into account the orientation information of the feature vector because, in the vascular region, the direction of the feature vector is usually approximate to the direction of the blood vessel, while in the non-vascular region, the direction of the feature vector is more random. Therefore, by calculating the direction Angle of the feature vector, the Frangi filter can further improve the detection accuracy of the vascular structure. Figure 3 shows the enhanced image using the noise-adaptive discrimination filtering algorithm based on grey relational analysis, where it is observed that the blood vessels are distinguishable and the background blood vessel contrast is improved. Figure 3a–e display the middle image of fundus image preprocessing.

### 2.5. Post-Processing

**Figure 4:**
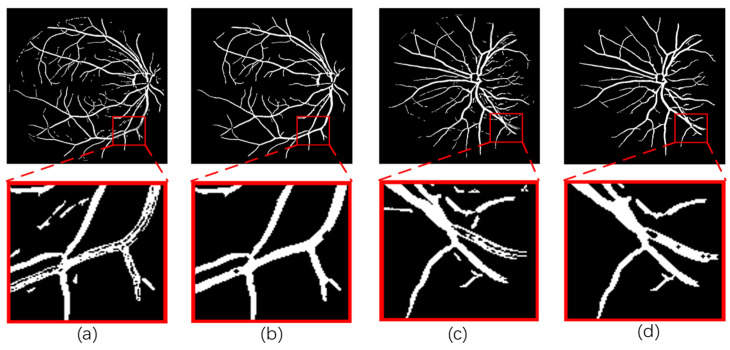
Images from the DRIVE dataset before and after post-processing: (**a**) and (**c**) are the images before post-processing. (**b**) and (**d**) are the images after post-processing.

The final segmentation results are obtained through the post-processing stage using morphological manipulation. First, a disc-shaped structural element is created for closure operations to fill the void in the blood vessel and connect the broken blood vessel, thereby improving the vascular connectivity. Subsequently, connected regions with fewer than 70 pixels are eliminated from the identified blood vessel regions, effectively removing erroneously detected isolated pixels and assisting in the removal of noise or pseudo-targets. Figure 4 shows a comparison of images from the DRIVE dataset before and after post-processing. (a) and (c) are the images before post-processing, and (b) and (d) are the images after post-processing. By enlarging the detailed image before post-processing, it can be found that several error detection pixels appear together with blood vessel pixels, and a large number of gaps exist. The post-processing operation effectively connects the disconnected blood vessel pixels and removes the error pixels, greatly improving the accuracy and connectivity of blood vessel segmentation.

## 3. Experimental Results and Discussion

### 3.1. Dataset Introduction

The datasets used in this study are DRIVE, STARE and HRF. These datasets are commonly used to evaluate retinal vessel segmentation in fundus images. The DRIVE dataset came from the Dutch Diabetes Screening Project, and for each image in the dataset, the corresponding blood vessel was manually segmented. The 40 images are equally divided into a training set and a test set, and because it is an unsupervised algorithm, the training set is not required. The STARE dataset contains 397 digital fundus images, of which only 20 had standard results, manually divided by two experts. Half of the fundus images contain pathological signs of different degrees, which poses a great challenge to the robustness and accuracy of the algorithm. The High-Resolution Fundus (HRF) image database has three sets of fundus images: healthy retinas, glaucoma-affected retinas, and diabetic retinopathy retinas. There are 45 pictures in total, and each set has 15 images. Each image has a resolution of 3504 × 2336 pixels and an 8-bit color depth per color plane. A binary mask and human-segmented blood vessel ground truth are provided for each image.

### 3.2. Evaluation Indicators

The proposed method evaluates the performance of the segmented output image by comparing it with the corresponding gold standard. Sensitivity (Se), Specificity (Sp), Accuracy (Acc) and Precision (Pr) were used to evaluate the validity of the algorithm. Accuracy represents the ratio of the number of pixels correctly segmented by the segmentation method to the number of pixels in the entire image. Accuracy is regarded as one of the most widely accepted measures for quantifying the effectiveness of segmentation results. Precision is the proportion of predicted positive classes in a classification problem that are actually positive. Another way to measure the model’s accuracy is through the F1-score. It is a harmonic average of accuracy and recall that strikes a balance between the two and is often used to assess the accuracy of a model, especially in unbalanced datasets. One more frequently used measure to evaluate the segmented result is the Jaccard coefficient (JC). It measures the percentage of overlap between the segmented output and the ground truth. These evaluation indicators are defined as follows:

(10) $$Se=\frac{T_{P}}{T_{P}+F_{N}}$$

(11) $$Sp=\frac{T_{N}}{T_{N}+F_{P}}$$

(12) $$Acc=\frac{T_{P}+T_{N}}{T_{P}+T_{N}+F_{P}+F_{N}}$$

(13) $$Pr=\frac{T_{P}}{T_{P}+F_{p}}$$

(14) $$F1-score=\frac{2\times (Se\times Pr)}{Se+Pr}=\frac{{2T}_{P}}{F_{P}+F_{N}+2T_{p}}$$

(15) $$JC=\frac{T_{P}}{T_{P}+F_{p}+F_{N}}$$

where $T_{P}$ is the number of true-positive samples, $T_{N}$ is the number of true-negative samples, $F_{P}$ is the number of false-positive samples, $F_{N}$ is the number of false-negative samples.

### 3.3. Visual Results

**Figure 5:**
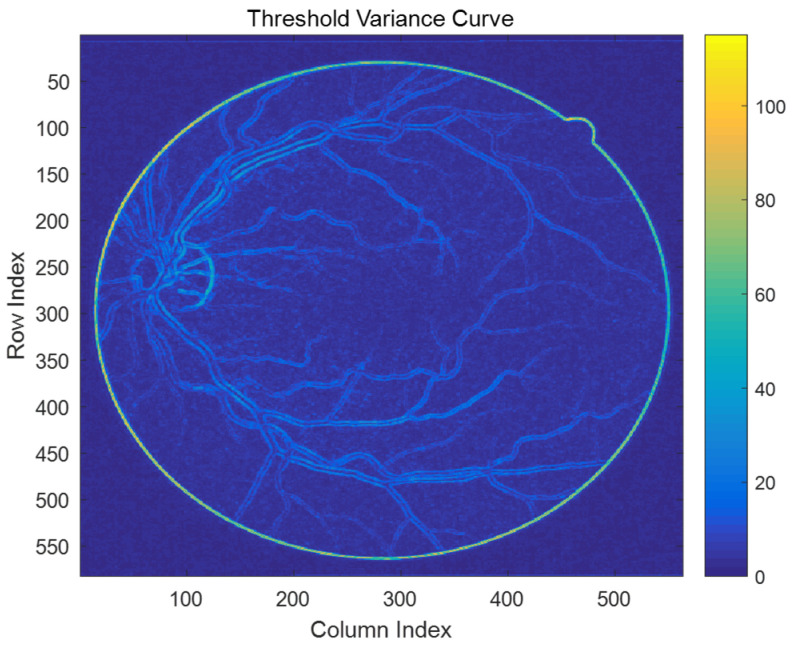
Threshold variation map of NADF-GRA.

The experiment was carried out on Intel Core-i7 processor with 8 GB of RAM and a Windows 10 operating system. The algorithm was completed in a Matlab R2016a operating environment. The algorithm processes each image with an average of approximately 3.5 s for preprocessing, 0.39 s for segmentation, and 1.21 s for post-processing. Thus, the entire process typically completes in about 5 s per image, demonstrating high efficiency for supervised algorithms. Figure 5 depicts the threshold variation map of the noise-adaptive discrimination filtering algorithm based on grey relational analysis (NADF-GRA). Each pixel in the image corresponds to the intensity of threshold variation within a local window of the original image. Warmer colors like red indicate higher threshold variations, whereas cooler colors like blue indicate lower variations. Typically, regions with higher threshold variations require larger filter sizes to maintain edge sharpness, while regions with smaller variations can use smaller filters to preserve more detail. Effectively adjusting the threshold strategy to match the characteristics of retina images involves selecting appropriate thresholds.

**Figure 6:**
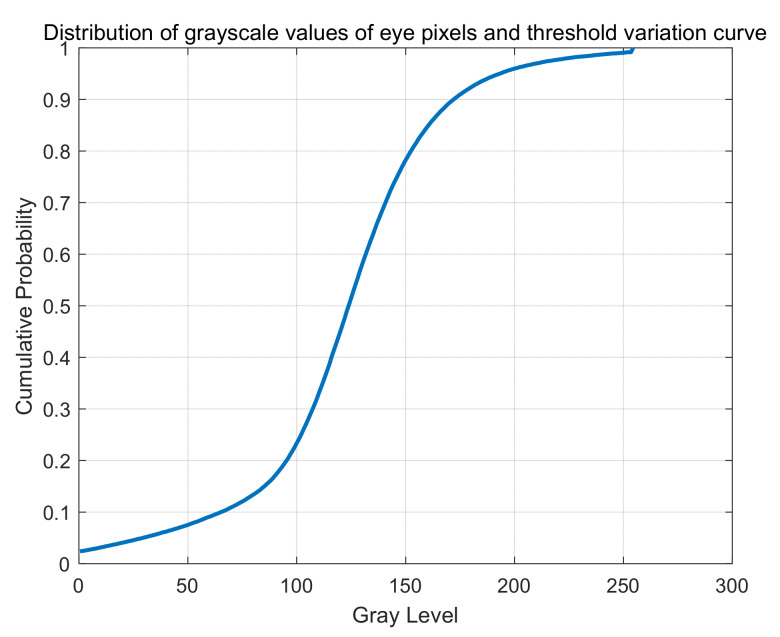
Threshold variation curve of TS-GRA.

Figure 6 shows the threshold variation curve of the threshold segmentation model based on grey relational analysis (TS-GRA). This curve illustrates the relationship between pixel grayscale levels and cumulative probability within the region of interest (retina). The horizontal axis represents grayscale levels from 0 to 255, and the vertical axis represents the cumulative probability, which denotes the percentage of pixels below each grayscale level. The optimal threshold typically resides at the steepest point of the curve, where the transition from foreground (retina) to background (surrounding area) is most pronounced. This point signifies a threshold that maximizes the segmentation accuracy between the eye and its surroundings, facilitating precise segmentation tasks.

**Figure 7:**
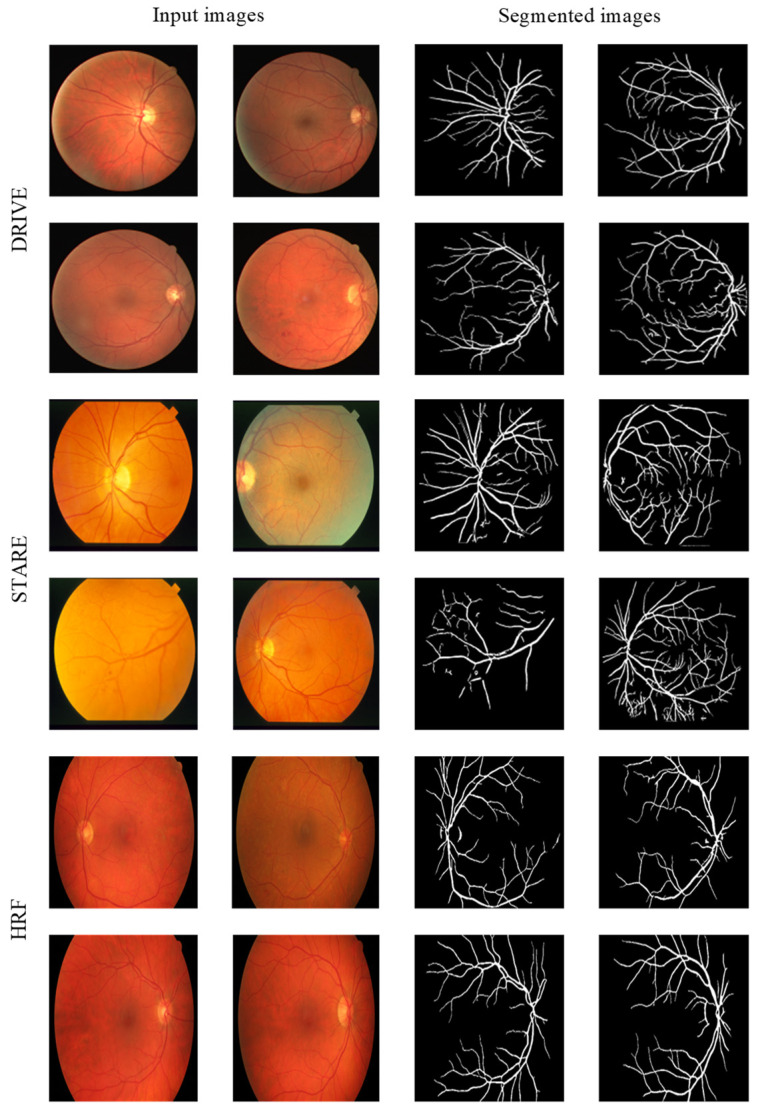
Segmentation results of the DRIVE, STARE and HRF datasets.

Figure 7 shows the visualized segmentation results of the proposed method on partial images from the DRIVE, STARE and HRF datasets.

**Figure 8:**
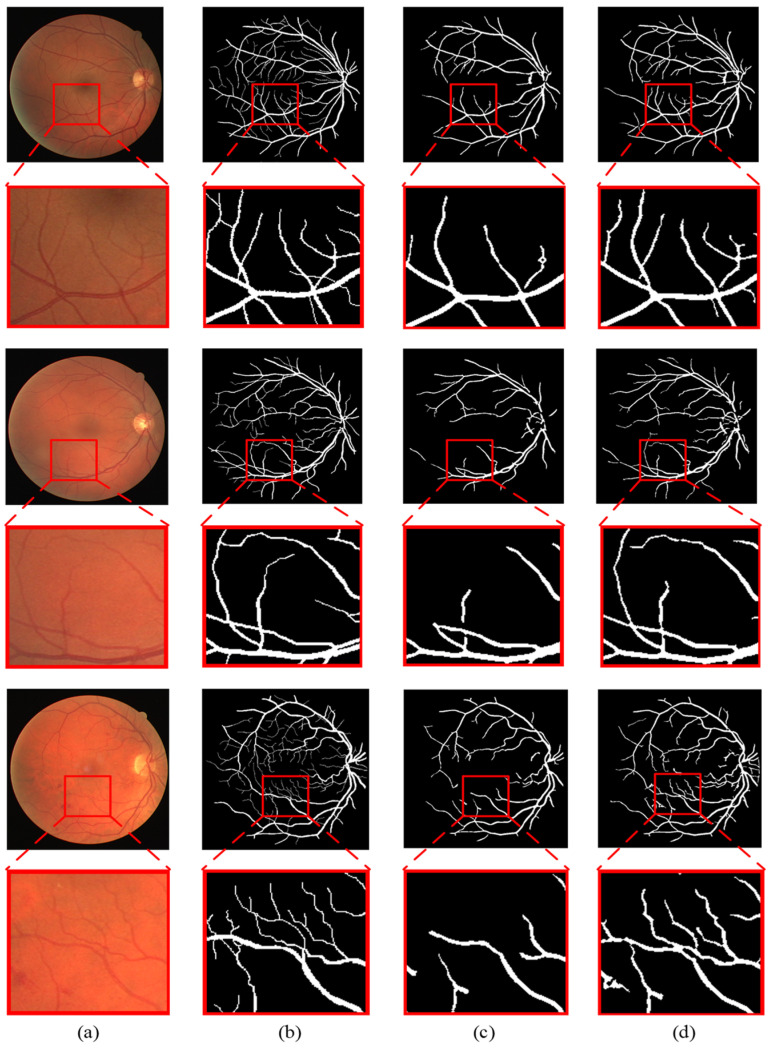
Magnification and comparison of segmentation results of different algorithms on DRIVE: (**a**) the original image, (**b**) the ground truth, (**c**) segmentation results under the traditional GLCM model, (**d**) segmentation results under the novel GLCAA model.

**Figure 9:**
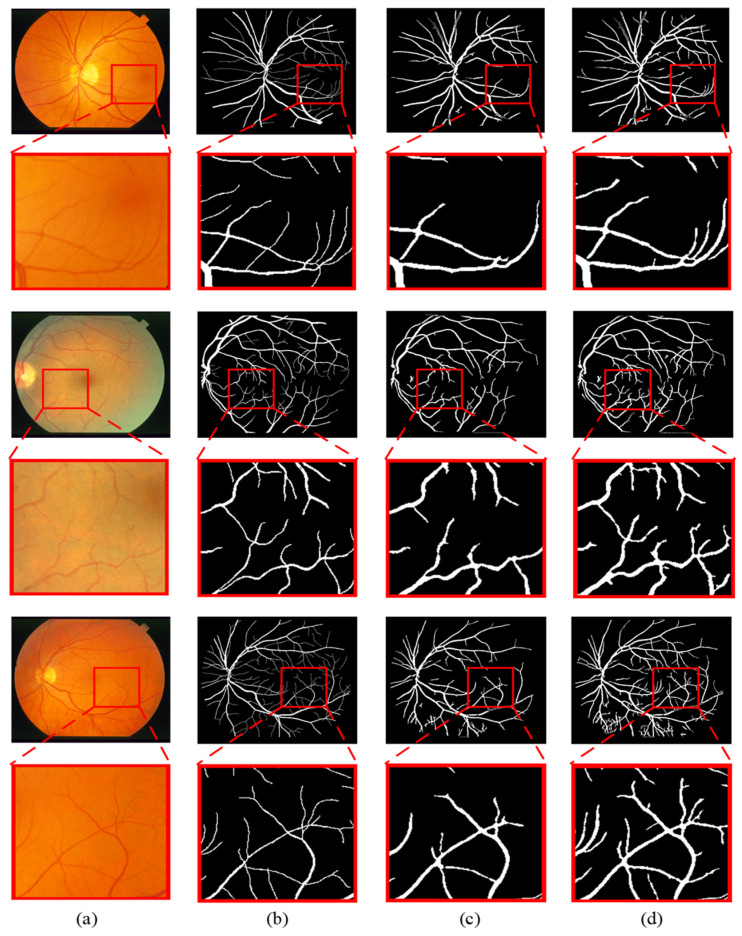
Magnification and comparison of segmentation results of different algorithms on STARE: (**a**) the original image, (**b**) the ground truth, (**c**) segmentation results under the traditional GLCM model, (**d**) segmentation results under the novel GLCAA model.

To better illustrate the advantages of the proposed method, the segmentation results under the ground truth, the traditional gray-level co-occurrence matrix (GLCM) model and the novel gray-level co-occurrence analysis adaptive (GLCAA) model are compared. Figure 8 and Figure 9 present enlarged comparisons of different models’ segmentation results of partial images from the DRIVE and STARE datasets. Panels (a)–(d), respectively, depict the original input image, the ground truth, segmentation results under the traditional GLCM model, and segmentation results under the novel GLCAA model. From the figures, it can be observed that in the segmentation results based on the traditional GLCM model, many thin blood vessels are not detected. In contrast, the segmentation results obtained using the proposed method closely resemble the ground truth, with many subtle thin blood vessels being detected. Additionally, for the STARE dataset, most retinal images contain varying degrees of pathological features. The proposed method accurately detects both thick and thin blood vessels while also effectively removing much of the noise interference.

### 3.4. Comparative Analysis of Objective Results

In order to further analyze the performance of the algorithm, these performance measures have been employed in this study. Although various performance indices are found in the literature, mostly Acc, Se, Sp are utilized for validation. Therefore, we present a comparative report of the proposed work and other different algorithms based on these parameters. Table 1 shows the results and average values of 20 images on DRIVE and STARE. Table 2 shows the results and average values of 20 images on STARE. Table 3 shows the results on HRF. Experimental results show that the average accuracy of the proposed method on DRIVE and STARE reaches 96.03% and 95.46%, respectively, which is superior to most other methods. The mean specificity was 98.51% and 97.85%, respectively, achieving the highest specificity on the DRIVE dataset. However, the lower sensitivity index may be due to the application of post-processing methods, which miss some very fine blood vessels in the segmented images, and these fine blood vessel structures highly overlap with the background and are therefore difficult to distinguish. Even with the image enhancement operation, these vessels showed a disconnected structure in the segmentation results, causing them to be incorrectly assumed to be noise and removed. Nevertheless, if the post-processing stage is omitted from the proposed framework, it may increase the sensitivity index but compromise the accuracy parameter.

Table 4 and Table 5 are the comparison of different experimental results of different algorithms on the DRIVE and STARE datasets respectively. Moreover, the Pr, F1-score and JC of the proposed work are ranked with some of the methods in Table 6 separately. The table shows a comparison between the proposed method and some supervised and unsupervised methods, as well as a comparison with the traditional grey relational degree algorithm. Although the proposed method belongs to the latter class of unsupervised methods, the results show that it is also superior to partially supervised algorithms. The tabular data show that the level set and region growth methods proposed in literature [15] have a poor segmentation effect on some abnormal retinal images, resulting in low accuracy. Literature [18] proposes some improved matching filter methods, which have low accuracy due to the fact that these methods use the same original filter parameters for all images, and the filter performance is uneven for images. The methods proposed in reference [19] achieve high sensitivity values in datasets. The use of Mamdani fuzzy rules (Type-2) for edge detection allows the algorithm to handle uncertainty and ambiguity in the image, leading to more accurate identification of blood vessel edges. And the algorithm employs the Green formula to calculate and exclude microaneurysms and other small-area formations from the final image, reducing false positives. However, the accuracy rate was only 86.5%. The lower accuracy may be influenced by some false positives. Due to the high sensitivity of the algorithm, it might interpret minor variations in grayscale as blood vessel edges, leading to the misclassification of non-vessel areas as vessels. The state-of-the-art supervised method proposed in reference [20] achieves high sensitivity values in datasets. MDUNet utilizes a multi-scale feature fusion technique, employs Dense Blocks to extract rich low-level features, and maintains high-resolution feature maps through an HR Block. This approach helps preserve more spatial information, thereby enhancing the model’s sensitivity. However, the accuracy of the method proposed in this paper is higher than that of this method on the DRIVE dataset. However, the accuracy of these methods is very poor compared to the proposed frameworks. And since accuracy is a balanced measure of correctly identifying blood vessels and background pixels, this method is considered superior among all methods. In addition, for the traditional grey relational degree model, the proposed method has improved on the three evaluation indexes, and for the DRIVE dataset, the accuracy, sensitivity and specificity have improved by 1.58%, 9.27% and 0.06% respectively. For the STARE dataset, the accuracy, sensitivity, and specificity improved by 0.9%, 7.48%, and 0.19%, respectively.

Table 6 compares Precision, F1-score, and JC with a few state-of-the-art approaches. The JC is found to be the highest as compared to the other methods for all the databases. The Noise-Adaptive Discrimination Filtering algorithm (NADF-GRA) effectively reduces the impact of noise and employs post-processing techniques such as cavity filling and removal of isolated pixels, thereby enhancing vessel connectivity. These are the reasons for the outstanding performance of the JC. Furthermore, since unsupervised methods do not rely on labeled data, they may be better at capturing the intrinsic structure of the data. Similarly, the Pr and F1-Score are the second contestants for both of the datasets. Table 7 shows the results of the ablation experiments of NADF-GRA and TS-GRA modules conducted on DRIVE and STARE, it can be observed that the method utilizing the traditional GLCM filtering algorithm and GLCM segmentation model achieved Acc, Se, and Sp metrics of 0.9445, 0.5936, and 0.9845 on DRIVE, respectively. After incorporating the NADF-GRA and TS-GRA modules, the metrics improved to 0.9603, 0.6863, and 0.9851, respectively. The Acc, Se and Sp indicators on STARE improved from 0.9456, 0.5957 and 0.9766 to 0.9546, 0.6705 and 0.9785. Ablation experiments show that our proposed module can improve the accuracy of retinal blood vessel segmentation, and the combination of the two is better. The Noise-Adaptive Discrimination Filtering algorithm (NADF-GRA) and the Threshold Segmentation Model (TS-GRA) help to improve performance indicators. These enhancements can be attributed to the NADF-GRA’s ability to effectively suppress noise while preserving vital vascular details and the TS-GRA’s precision in identifying the vascular edges by leveraging grey relational analysis. When applied to the STARE dataset, the method further demonstrated its robustness. The lower sensitivity increase in comparison to the DRIVE dataset may be due to the more complex nature of the STARE images, which include a variety of pathological features that can obscure fine blood vessels.

## 4. Conclusions

In this paper, a novel grey relational-based model for retinal vessel segmentation is proposed. The reason for using this method is that the gray system theory is proposed to solve the problem of “few data uncertainties”, and the retinal blood vessel images are small in a number of samples and there are uncertainties. The core of this method is to measure the similarity between the reference sequence and the comparison sequence by grey relational analysis. Due to the discontinuity in the distribution of retinal blood vessels and the uncertainty in the system, a new grey relational adaptive discriminant filtering algorithm is proposed, and a good filtering effect is obtained. In addition, the grayscale of the pixel mutation in the edge part of the blood vessel generally satisfies the continuity in a certain direction or texture shape, which is very different from the grayscale mutation caused by noise. Therefore, the gray relational degree is used to segment the blood vessel, thus achieving more accurate results, and many fine blood vessels can also be well detected. The proposed method performs well in terms of connectivity between retinal blood vessels and is also easier to implement. Because some very fine blood vessels are misclassified as background, which will lead to poor results to some extent, we will further study finer granularity segmentation.

## Figures and Tables

**Table 1:** Performance evaluation on DRIVE.

Image	DRIVE					
	Acc	Se	Sp	Pr	F1-Score	JC
1	0.9602	0.7975	0.9762	0.7661	0.7815	0.6414
2	0.9594	0.7793	0.9794	0.8122	0.7954	0.6604
3	0.9533	0.6288	0.9892	0.866	0.7386	0.5730
4	0.9617	0.6914	0.9891	0.8654	0.7687	0.6243
5	0.9582	0.6267	0.9925	0.8961	0.7475	0.5842
6	0.9545	0.5924	0.9936	0.9084	0.7571	0.5590
7	0.9516	0.6481	0.9821	0.7845	0.7398	0.5501
8	0.9524	0.5656	0.9888	0.8261	0.6715	0.5054
9	0.9693	0.5914	0.9895	0.8329	0.6917	0.5287
10	0.9627	0.6600	0.9898	0.8533	0.7543	0.5927
11	0.9509	0.7143	0.9742	0.7311	0.7596	0.5657
12	0.9699	0.6945	0.9847	0.8104	0.7480	0.5975
13	0.9551	0.6584	0.9872	0.8482	0.7493	0.5890
14	0.9624	0.7489	0.9811	0.7774	0.7729	0.6167
15	0.9547	0.6947	0.9747	0.6795	0.7170	0.5233
16	0.9641	0.7086	0.9841	0.8155	0.7583	0.6107
17	0.9609	0.6528	0.9893	0.8495	0.7483	0.5852
18	0.9638	0.7622	0.9811	0.7763	0.7792	0.6249
19	0.9723	0.7898	0.9888	0.8640	0.8253	0.7025
20	0.9676	0.7200	0.9873	0.8176	0.7657	0.6204
Average	0.9603	0.6863	0.9851	0.8190	0.7535	0.5928

**Table 2:** Performance evaluation on STARE.

Image	STARE					
	Acc	Se	Sp	Pr	F1-Score	JC
1	0.9338	0.6070	0.9622	0.6578	0.6981	0.5266
2	0.9328	0.4602	0.9629	0.6172	0.6663	0.5394
3	0.9243	0.7826	0.9449	0.627	0.6922	0.5091
4	0.9444	0.4150	0.9980	0.9176	0.6326	0.526
5	0.9593	0.7758	0.9787	0.6237	0.6926	0.5297
6	0.9453	0.4931	0.9821	0.6859	0.6541	0.5934
7	0.9481	0.6948	0.9620	0.7282	0.7391	0.589
8	0.9521	0.6011	0.9735	0.6961	0.7377	0.5705
9	0.9595	0.6815	0.9715	0.7619	0.6912	0.5219
10	0.9584	0.5784	0.9793	0.7645	0.6545	0.5336
11	0.9591	0.8693	0.9820	0.7294	0.7971	0.547
12	0.9712	0.8789	0.9721	0.6686	0.7538	0.6048
13	0.9495	0.7987	0.9859	0.6792	0.7401	0.5968
14	0.9597	0.7791	0.9764	0.6993	0.7534	0.5307
15	0.9692	0.7874	0.9823	0.7887	0.7639	0.618
16	0.9591	0.7057	0.9935	0.8522	0.6614	0.5902
17	0.9530	0.6951	0.9857	0.7529	0.7975	0.5717
18	0.9761	0.6270	0.9947	0.8588	0.7272	0.5713
19	0.9765	0.6082	0.9930	0.7946	0.7016	0.5403
20	0.9612	0.5709	0.9891	0.7706	0.6632	0.4961
Average	0.9546	0.6705	0.9785	0.7337	0.7109	0.5553

**Table 3:** Performance evaluation on HRF.

Image	HRF								
	H (%)			DR (%)			G (%)		
	Pr	F1-Score	JC	Pr	F1-Score	JC	Pr	F1-Score	JC
1	81.28	81.57	69.03	76.23	72.46	60.56	72.63	72.90	54.32
2	79.50	80.29	68.85	76.59	69.07	58.93	72.17	71.75	52.12
3	78.99	79.92	49.89	69.22	66.23	53.06	73.69	70.90	50.89
4	80.99	81.02	59.89	76.88	73.74	52.69	71.61	70.91	51.41
5	82.77	75.97	63.49	76.84	74.54	57.41	72.74	69.89	51.58
6	79.89	76.20	65.48	76.19	69.49	58.44	70.40	69.63	52.96
7	82.96	82.80	69.93	76.15	75.78	59.13	72.11	70.88	50.12
8	81.64	78.26	67.16	77.82	78.45	56.33	70.88	70.79	50.58
9	80.67	76.79	69.76	79.16	69.89	60.75	69.37	69.88	49.47
10	78.91	80.21	68.23	78.61	64.36	57.55	67.81	69.10	50.25
11	80.65	78.31	62.60	77.13	79.81	59.86	70.52	71.35	52.77
12	83.70	78.71	65.55	75.06	78.85	58.08	68.93	71.83	50.96
13	79.93	76.15	59.85	79.29	76.30	60.71	69.03	70.85	50.98
14	78.18	78.68	64.94	76.71	75.44	58.29	71.68	70.67	48.52
15	79.35	78.44	68.54	74.24	72.51	55.42	69.56	69.72	50.63
avg	80.63	78.89	64.88	76.41	73.12	57.81	70.88	70.74	51.17

**Table 4:** Comparison of experimental results of different algorithms on DRIVE.

Methods		Acc	Se	Sp
**Supervised methods**	Tang et al. [2]	0.9574	0.8083	0.9796
	Guo S [3]	0.9575	0.7993	0.9806
	Dong et al. [21]	0.9586	0.7954	—
	Qu et al. [22]	0.9629	0.8749	0.9758
	Jayachandran [20]	0.9587	0.8072	0.9803
	Zhao et al. [9]	0.9580	0.7740	0.9790
**Unsupervised methods**	Odstrcilik et al. [23]	0.9340	0.7060	0.9693
	Roy et al. [24]	0.9295	0.4392	0.9622
	Yang et al. [25]	0.9522	0.7181	0.9747
	Nath et al. [18]	0.9493	0.4304	0.9024
	Tian et al. [26]	0.9554	0.6942	0.9802
	Huang et al. [27]	0.9535	0.6650	0.9812
	Mahapatra et al. [28]	0.9605	0.7020	0.9844
	Shukla et al. [29]	0.9476	0.7015	0.9836
	Traditional GLCM model	0.9445	0.5936	0.9845
	Proposed method	0.9603	0.6863	0.9851

**Table 5:** Comparison of experimental results of different algorithms on STARE.

Methods		Acc	Se	Sp
**Supervised methods**	Vega et al. [30]	0.9189	0.8179	0.9269
	Huang et al. [4]	0.9728	0.8304	0.9862
	Qu et al. [22]	0.9724	0.8852	0.9820
	Jayachandran [20]	0.9694	0.9836	0.8213
	Zhao et al. [9]	0.9570	0.7880	0.9760
**Unsupervised methods**	Lazar and Hajdu [31]	0.9492	0.7248	0.9751
	Ramos et al. [32]	0.9231	0.7116	0.9454
	Roy et al. [24]	0.9488	0.4317	0.9718
	Orujov et al. [19]	0.8650	0.8342	0.8806
	Yang et al. [25]	0.9513	0.6713	0.9731
	Tian et al. [26]	0.9492	0.7019	0.9771
	Huang et al. [27]	0.9537	0.7273	0.9622
	Mahapatra et al. [28]	0.9601	0.6846	0.9802
	Traditional GLCM model	0.9456	0.5957	0.9766
	Proposed method	0.9546	0.6705	0.9785

**Table 6:** Comparison using more performance parameters.

Methods	Database	Pr	F1-Score	JC
Vega et al. [30]	DRIVE	64.02	68.84	-
Nath et al. [18]	DRIVE	45.11	44.05	28.37
Orujov et al. [19]	DRIVE	34.02	38.00	55.00
	STARE	70.15	53.35	36.17
Annunziata et al. [33]	STARE	83.31	76.82	-
Ramos et al. [32]	DRIVE	68.80	73.35	-
Shukla et al. [29]	DRIVE	51.94	59.69	-
	STARE	46.38	55.87	-
Mahapatra et al. [28]	DRIVE	81.24	75.31	58.97
	STARE	74.40	71.29	55.66
	HRF(H)	80.62	78.78	64.51
	HRF(DR)	77.32	73.86	58.61
	HRF(G)	70.28	70.70	51.14
Traditional GLCM model	DRIVE	80.49	74.01	58.32
	STARE	72.46	70.53	54.27
	HRF(H)	79.91	78.10	63.58
	HRF(DR)	75.69	72.27	56.79
	HRF(G)	70.02	69.80	50.52
Proposed method	DRIVE	81.90	75.35	59.28
	STARE	73.37	71.09	55.53
	HRF(H)	80.63	78.89	64.88
	HRF(DR)	76.41	73.12	57.81
	HRF(G)	70.88	70.74	51.17

**Table 7:** Ablation experiment on DRIVE and STARE.

Method	Filtering Method		Segmentation Method		DRIVE			STARE		
	GLCM Filtering	NADF-GRA	GLCM Segmentation	TS-GRA	Acc	Se	Sp	Acc	Se	Sp
Method 1	√	–	√	–	0.9445	0.5936	0.9845	0.9456	0.5957	0.9766
Method 2	√	–	–	√	0.9484	0.5952	0.9844	0.9504	0.6499	0.9764
Method 3	–	√	√	–	0.9569	0.6019	0.9849	0.9528	0.6681	0.9780
Method 4	–	√	–	√	0.9603	0.6863	0.9851	0.9546	0.6705	0.9785

## Data Availability

The data analyzed during the current study are available from the corresponding author upon reasonable request.
